# Supplementary figures and images for: Is Nutritional Ultrasound as Useful and Accurate as Computed Tomography to Assess Sarcopenia in Cancer Patients? A Systematic Review
Source: Cancers (Basel). 2025 Nov 18;17(22):3683. doi: 10.3390/cancers17223683 (PMC12651387; doi:10.3390/cancers17223683)

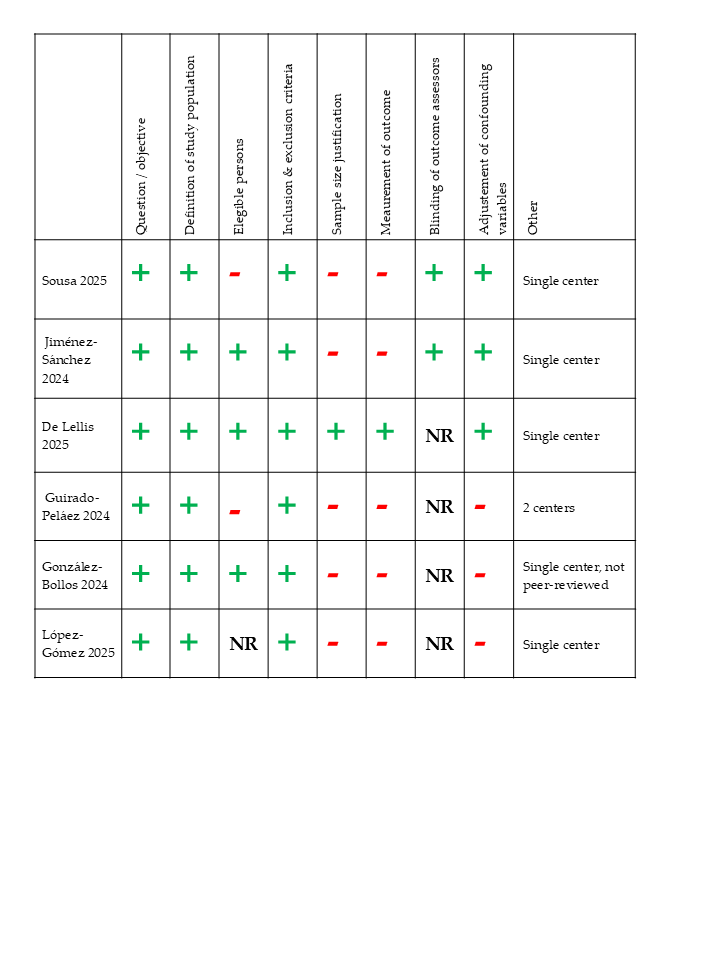

Supplement: Supplementary file 1 [file cancers-17-03683-s001.zip › cancers-3965103-supplementary Figure S1.tif]
